# First- and second-order social contact network structure in southern China

**DOI:** 10.1098/rsif.2025.0232

**Published:** 2025-11-26

**Authors:** Claire Perrin Smith, Jonathan Michael Read, Steven Riley, Derek Cummings, Kin On Kwok, Chao Qiang Jiang, Justin Lessler

**Affiliations:** ^1^ Department of Epidemiology, The University of North Carolina at Chapel Hill Gillings School of Global Public Health, Chapel Hill, NC, USA; ^2^ Department of Lancaster Medical School, Lancaster University, Lancaster, UK; ^3^ MRC Centre for Global Infectious Disease Analysis, Imperial College London, London, UK; ^4^ Department of Epidemiology, Johns Hopkins Bloomberg School of Public Health, Baltimore, MD, USA; ^5^ JC School of Public Health and Primary Care, The Chinese University of Hong Kong, Hong Kong; ^6^ Stanley Ho Centre for Emerging Infectious Diseases, The Chinese University of Hong Kong, Hong Kong; ^7^ Guangzhou Twelfth People's Hospital, Guangzhou, Guangdong, People’s Republic of China

**Keywords:** contact, network structure, clustering, transmission

## Abstract

The contact network structure resulting from social interaction between people is a key aspect of epidemic dynamics and control. While many studies have measured first-order network characteristics such as degree, measuring higher order properties of these networks, such as clustering, remains a challenge. Here, we present the results of a study of first- and second-order network structure from a representative cohort of individuals in Guangdong province, China. The number of reported daily contacts is similar across individuals aged 2 to 55 years, except for young adults (ages 16–25) who have relatively fewer daily contacts, while the number of contacts declines with age above 55 years old. The association between age and contact rate persisted after adjusting for mediating factors. Individuals living in higher population density areas made more contacts outside the home than individuals in low-density areas. Contacts of young children and older adults were more locally clustered than middle-aged adults. Individuals living in high population density areas had lower levels of local clustering compared with individuals from low-density areas. Adjustment for characteristics of the contacts themselves reduces the variation in local clustering between participants of different ages; however, the strong association with population density remains.

## 1. Introduction

Recommendations for non-pharmaceutical interventions, characterization of community groups at high risk for super-spreading events and mathematical modelling of disease spread all rely on underlying assumptions about social contact [1,2]. Contact networks, where individuals are depicted as nodes and their contacts as links between nodes, are a key tool for characterizing patterns of interaction and their impact on epidemic dynamics [3]. Because respiratory pathogens, such as influenza or SARS-CoV-2, are spread predominantly through close interpersonal contacts [4], measuring the networks formed by such interactions is essential for understanding and predicting the spread of these diseases.

Fully enumerated contact networks, where all links that could potentially result in pathogen transmission between individuals are known, provide the most insight into overall network structure. Such detailed contact data, however, are all but impossible to collect for most infectious diseases and settings and are usually limited to smaller, well-defined communities or limited definitions of contact. In the case of small, closed populations, the use of wearable proximity sensors has allowed for a detailed description of face-to-face contact patterns in settings such as schools [5] and hospitals [6]. Focusing on a narrow subset of contacts, such as sexual encounters, also facilitates a more complete characterization of contact networks. For example, it was possible to enumerate a nearly complete network of sexual contacts in the community of Likoma Island, Malawi, enabling researchers to understand variation in HIV prevalence throughout the network [7].

Characterizing the distribution of contacts in the general population relevant to respiratory pathogen transmission is difficult given the number and heterogeneity of such contacts. The definition of a contact relevant for transmission may even vary between respiratory pathogens (e.g. influenza and measles), depending on the specifics of their transmission dynamics [4,8]. Despite these uncertainties, efforts have been made to capture critical properties of these networks for face-to-face interactions, the contacts that are probably most important to respiratory pathogen transmission. Many studies have used surveys of contact behaviour to collect information on the number (degree) and nature of face-to-face interactions individuals have over some period of time [9–12]. This provides information on ‘first-order’ network properties, such as the degree distribution of individual nodes in the network (i.e. the distribution of the number of direct contacts). However, this approach provides limited information on the structure of the overall network.

To understand how pathogens might spread among populations, it is often useful to characterize the interconnectedness of an individual’s contacts [13]. ‘Second-order’ network properties, which encompass both the interconnectedness of an individual’s first-order contacts and the broader structure of the network in which that individual resides, have been shown to have implications for pathogen spread. Clustering (also termed transitivity), in particular, is a second-order network property that is an important driver of epidemic dynamics. In general, higher levels of clustering tend to decrease both the overall speed of an epidemic and the probability of a large final outbreak size [14–16], although this may vary if other network properties differ, particularly the variance in the degree distribution [14,15,17,18]. Simulation studies have shown that when clustering and degree heterogeneity increase simultaneously, epidemics can take off more quickly [14,15]. Additionally, network clustering has important consequences for pathogen evolution and infection control efforts [19,20].

A particularly important driver of heterogeneity in contact network structure is age. Prior work has shown that there is considerable variability in both the number and type of contacts made by individuals of different ages [11,21], leading some age groups to have a disproportionate impact on epidemic dynamics. For example, school-aged children tend to have higher numbers of daily contacts than adults [9,11,12], and schools have been repeatedly shown to play an important role in driving epidemics [22]. Additionally, contacts tend to be assortative by age, with individuals more likely to interact with others in the same age group [11]. The degree to which contacts are assortative has also been shown to vary with age, with the youngest and oldest age groups having the most pronounced assortative mixing rates [23]. The relationship between age and contact network structure is probably mediated by factors such as occupational status, household structure and community type.

A more detailed understanding of local contact network structure is essential for a more accurate modelling of transmission dynamics and better characterization of groups at heightened risk for pathogen introduction and spread. Here we use contact survey data from the Fluscape study to examine variations in local contact networks over age and location in southern China, and what factors mediate these relationships.

## 2. Methods and material

### 2.1. Study population

The Fluscape cohort included 40 communities sampled along a spatial transect extending from Guangzhou city centre, with target enrolment of 20 households per community. In cases where households withdrew from the study, replacements were introduced in subsequent visits. Efforts were made by study staff to enrol all residents aged 2 years or older within each household. Overall, 3454 unique individuals from 1251 households participated in the Fluscape study across visits 1 to 5. The present analysis focuses on the subset of 3184 participants who participated in study visits 2–5 and for whom complete contact and participant surveys are available. For more information on the Fluscape study, refer to Jiang *et al.* [24].

The Fluscape study was approved by institutional review boards of Johns Hopkins Bloomberg School of Public Health, University of Florida, University of Liverpool, University of Hong Kong, Guangzhou No. 12 Hospital and Shantou University.

### 2.2. Data collection

Five study visits were completed from 2009 to 2017, with visits spaced one to two years apart. At each visit, three survey instruments were administered via face-to-face interviews (the household, participant and contact surveys). The household survey collected information on household composition, animal ownership and travel history of household members. The participant survey collected information on individual sociodemographic characteristics and health status. The contact survey asked participants to report about all social interactions from the prior day with whom they had a face-to-face conversation or which involved touch.

Contacts could be reported individually or as groups (e.g. everyone a cafeteria worker serves in a day). Each entry in the contact survey, whether an individual or a group, is defined as a single ‘contact event’. Each contact event has an associated group size (one for individual contacts), and the sum of these group sizes gives each participant’s total number of reported contacts. Individuals included in multiple contact events were flagged as repeats by participants on the contact survey and were only counted once when calculating the total number of reported contacts. For each contact event, the participant was asked its location, setting (home, work, school, social or other), duration and whether it involved touch. Participants were also asked the frequency with which they contacted those involved in the event and their age(s).

The contact survey also attempted to measure the connectivity between contacts. For each participant, two contact events were randomly selected from all reported contacts (the ‘randomly selected primary contacts’). The participant was asked how likely it was that each randomly selected primary contact also interacted with each of their other reported contacts (the ‘secondary contact’) during the week prior, with participants able to respond ‘yes’, ‘probably yes’, ‘probably no’ or ‘no’. In the main analysis here, responses were dichotomized, with ‘yes’ and ‘probably yes’ grouped together as an affirmative response. Sensitivity analyses were conducted to assess the impact of different certainty cut-offs for an affirmative response (electronic supplementary material, tables S7 and S8).

Participants aged 16–75 were categorized into 10 year age groups. Children aged 2–15 were categorized together due to the low number of participants under age 5. Likewise, participants aged 76 and older were grouped together. Occupation status was categorized as ‘employed’, ‘student’ or ‘other’, with ‘other’ including children not in school and participants who reported being unemployed or retired. Population density at each household location was obtained from LandScan [25], and defined as the average of the estimated density of the square-kilometre tile that included the household and the eight bordering tiles in the year of the study visit. When including population density in hierarchical models, the population density at the first analysed study visit was used as a household-level covariate.

Respondents were asked to categorize contacts into one (or more in the case of groups) of five age groups: 0–4, 5–19, 20–39, 40–64 and 65 and over. Starting at the third visit, participants could also give a guess of the contact’s exact age. In the absence of a response for the age categorization, the age guess was used to infer the age category. The setting in which reported contacts were made was categorized as home, work, school, social or other. The social category included contacts reported as occurring in a location where the participant went to eat or drink, play games or sport or to see someone. Contacts reported as occurring on transportation or while shopping were classified as other. Respondents also reported the number of times per week they met a reported contact (less than once per week, 1–3 times per week or 4+ times per week) and the duration of the contact event (less than 10 min, 10–59 min or 60+ min), both of which are measures of contact intimacy. The total amount of time that a participant spends with a contact each week quantifies where a contact falls on the intimacy spectrum and is calculated as contact frequency multiplied by duration, with the values for each set to the midpoint of the category.

### 2.3. Statistical analysis

Uni- and multi-variate hierarchical quasi-Poisson regression models [26] were fitted to the number of reported contacts. All models included random effects at location (village or neighbourhood), household, participant and observation levels, with the observation-level random effects capturing the overdispersion in the number of daily reported contacts (i.e. making this quasi-Poisson regression). The study visit was considered a fixed effect. Household, participant and observation-level covariates were considered in analyses.

Each combination of a participant, a randomly selected primary contact and a secondary contact forms a potential contact triangle. The contact triangle is ‘connected’ if the participant reports that contact probably occurred between the random primary contact and the secondary contact in the prior week. Triangle connectedness is one measure of local clustering, or the degree to which the people connected to an individual in a network are also connected to each other. Hierarchical logistic regression models were fit to this data to estimate how contact-, individual- and household-level factors were associated with the probability of triangle connection. All models included random effects at the location (village or neighbourhood), household and participant levels. The study visit was considered a fixed effect.

Because both primary and secondary contacts could be groups, there are instances where a single potential contact triangle could represent multiple possible underlying contact triangles involving individual members of the group(s). In instances where two groups are selected, the number of underlying triangles can be very large (e.g. there would be 100 possible underlying triangles for two groups of 10). If the participant reports a connected contact triangle involving a group, the likelihood contribution for this observation is the probability that at least one of the underlying contact triangles within this observation is connected. Similarly, if the participant reports that such an observation is not connected, the likelihood contribution is the probability that none of the underlying contact triangles are connected, see electronic supplementary material, Methods. Observations in the top 0.5% of the number of individual triangles embedded within a group observation were trimmed prior to the analysis due to the undue influence these large sets of possible triangles could have on the likelihood. Further, in analyses which adjusted for contact characteristics, the possible different nature of group contacts was accounted for by including an indicator of group status and the number of possible underlying triangles as covariates.

Three nested models were fit to the data (electronic supplementary material, table S3). The ‘participant-only model’ included only characteristics of the study participant and their household. The ‘individual contact model’ included participant- and household-level covariates as well as the characteristics of each of the two contacts in the potential contact triangle. The ‘shared contact model’ included all participant-, household- and contact-level covariates as well as indicators for the relationship between the two contacts (e.g. whether both contacts occurred in the same setting). Models were compared using the Watanabe–Akaike information criterion (WAIC).

All models were run in Stan [27] and are fit using four Markov chain Monte Carlo (MCMC) chains with 2000 iterations each, with the first 1000 iterations discarded as warm-up. Convergence was assessed using the Gelman–Rubin statistic [28] and visualization of the trace plots. We had high confidence that the last 1000 iterations gave a high effective sample size for all results presented here, with the bulk and tail effective sample sizes for coefficients exceeding 200 in all models (electronic supplementary material, table S9). Model estimates are the mean of the posterior sampling distribution. All other analysis was done in R v. 4.1.3.

## 3. Results

The Fluscape study was a longitudinal cohort conducted in Guangdong, a province of southern China, which spanned from 2009 to 2017 and involved five complete visits [24]. There were 3184 unique individuals who participated in visits 2–5 of follow-up (2nd visit: 2017; 3rd: 2025; 4th: 2043; 5th: 1819) with 924 individuals participating in all four visits. Participants were 51% male and 43% lived in lower population density areas (less than 500 people per square kilometre). At baseline, 51% were employed and 13% were students, with the remainder being unemployed, retired, children not in school or of unknown occupation status (table 1). The age distribution of participants is generally reflective of the age distribution of the local population, with the exception of the youngest age groups, which are under-represented [24].

**Table 1.** Number of unique participants and participant visits stratified by participant characteristics.

	unique participants	participant visits	participant visits stratified by number of reported contacts			
	(*n* = 3184)	(*n* = 7904)	1–5 (*n* = 1947)	6–10 (*n* = 2134)	11–15 (*n* = 1377)	16+ (*n* = 2446)
age						
2–15	346 (11%)	684 (9%)	102 (5%)	198 (9%)	117 (8%)	267 (11%)
16–25	427 (13%)	733 (9%)	181 (9%)	207 (10%)	123 (9%)	222 (9%)
26–35	420 (13%)	953 (12%)	140 (7%)	251 (12%)	178 (13%)	384 (16%)
36–45	529 (17%)	1283 (16%)	234 (12%)	321 (15%)	239 (17%)	489 (20%)
46–55	566 (18%)	1605 (20%)	336 (17%)	418 (20%)	296 (21%)	555 (23%)
56–65	479 (15%)	1383 (17%)	401 (21%)	376 (18%)	250 (18%)	356 (15%)
66–75	258 (8%)	797 (10%)	300 (15%)	254 (12%)	120 (9%)	123 (5%)
75+	145 (5%)	436 (6%)	231 (12%)	105 (5%)	52 (4%)	48 (2%)
unknown	14 (0%)	30 (0%)	22 (1%)	4 (0%)	2 (0%)	2 (0%)
sex						
male	1628 (51%)	4039 (51%)	950 (49%)	1069 (50%)	717 (52%)	1098 (45%)
female	1541 (48%)	3834 (49%)	973 (50%)	1062 (50%)	658 (48%)	1346 (55%)
unknown	15 (0%)	31 (0%)	24 (1%)	3 (0%)	2 (0%)	2 (0%)
population per sq. km						
≤100	198 (6%)	435 (6%)	133 (7%)	116 (5%)	76 (6%)	110 (4%)
101–500	1175 (37%)	2882 (36%)	735 (38%)	808 (38%)	490 (36%)	849 (35%)
501–5000	1329 (42%)	3379 (43%)	748 (38%)	914 (43%)	627 (46%)	1090 (45%)
5000+	358 (11%)	1072 (14%)	300 (15%)	258 (12%)	161 (12%)	353 (14%)
unknown	124 (4%)	136 (2%)	31 (2%)	38 (2%)	23 (2%)	44 (2%)
occupation status						
employed	1608 (51%)	4107 (52%)	745 (38%)	1009 (47%)	751 (55%)	1602 (65%)
student	425 (13%)	831 (11%)	154 (8%)	226 (11%)	132 (10%)	319 (13%)
unemployed or retired	1138 (36%)	2934 (37%)	1025 (53%)	895 (42%)	492 (36%)	522 (21%)
unknown	13 (0%)	32 (0%)	23 (1%)	4 (0%)	2 (0%)	3 (0%)

Number of unique participants and participant visits stratified by age, sex, population density and occupation status at first visit. Participant visits are further stratified by the number of contacts reported at the visit. Column percentages may not sum to exactly 100% due to rounding error.

Across all follow-up visits, participants reported 39 386 contact events, 33% (12 969) of which were group contacts, representing 145 660 total contacts. Participants reported a median of 10 daily contacts in any given visit. The age distribution of reported daily contacts roughly mirrors the age distribution of participants, with approximately two-thirds of contacts in the 20–64 age range (excluding group contacts with multiple age groups). The majority (57%) of contact events occurred in the home (electronic supplementary material, table S1), but the majority of contacts (81%) were extra-household (table 2). Individuals aged 5–19 make the majority (59%) of their contacts at school, while working-age adults make the majority (56% for ages 20–39, 52% for ages 40–64) of their contacts in the workplace. Adults aged 65 and older make most of their contacts at home (31%) or in social (35%) and other (25%) settings. Nineteen per cent of reported contacts involved touch. The majority of contacts (73%) were with individuals the participant reported contacting four or more times per week. Likewise, most (51%) contacts were of long duration, lasting for an hour or more.

**Table 2.** Characteristics of reported contacts by age group.

		age group					
	total(*n* = 145 660)	2–4(*n* = 152)	5–19(*n* = 15 185)	20–39(*n* = 38 098)	40–64(*n* = 78 911)	65+(*n* = 13 199)	unknown(*n* = 115)
group size							
1	26 417 (18%)	101 (66%)	4003 (26%)	8054 (21%)	10 780 (14%)	3445 (26%)	34 (30%)
2–5	25 652 (18%)	32 (21%)	2527 (17%)	5463 (14%)	13 555 (17%)	4042 (31%)	33 (29%)
6–10	23 838 (16%)	19 (13%)	2144 (14%)	6329 (17%)	12 881 (16%)	2417 (18%)	48 (42%)
11–20	15 559 (11%)	0 (0%)	1830 (12%)	4595 (12%)	7948 (10%)	1186 (9%)	0 (0%)
21+	54 194 (37%)	0 (0%)	4681(31%)	13 657 (36%)	33 747 (43%)	2109 (16%)	0 (0%)
contact age							
0–4	2732 (2%)	8 (5%)	196 (1%)	1119 (3%)	1208 (2%)	196 (1%)	5 (4%)
5–19	15 238 (10%)	24 (16%)	9515 (63%)	2060 (5%)	2911 (4%)	714 (5%)	14 (12%)
20–39	21 040 (14%)	58 (38%)	1953 (13%)	9539 (25%)	8146 (10%)	1309 (10%)	35 (30%)
40–64	29 488 (20%)	32 (21%)	1541 (10%)	4992 (13%)	19 238 (24%)	3638 (28%)	47 (41%)
65+	5146 (4%)	8 (5%)	436 (3%)	694 (2%)	1665 (2%)	2342 (18%)	1 (1%)
multiple	70 918 (49%)	21 (14%)	1408 (9%)	19 530 (51%)	45 430 (58%)	4516 (34%)	13 (11%)
unknown	1098 (1%)	1 (1%)	136 (1%)	164 (0%)	313 (0%)	484 (4%)	0 (0%)
contact setting							
home	27 753 (19%)	88 (58%)	3235 (21%)	7514 (20%)	12 825 (16%)	4047 (31%)	44 (38%)
work	63 756 (44%)	0 (0%)	447 (3%)	21 289 (56%)	40 993 (52%)	1008 (8%)	19 (17%)
school	9235 (6%)	18 (12%)	8970 (59%)	64 (0%)	27 (0%)	150 (1%)	6 (5%)
social	25 234 (17%)	39 (26%)	1625 (11%)	4958 (13%)	13 901 (18%)	4675 (35%)	36 (31%)
other	19 677 (14%)	7 (5%)	908 (6%)	4270 (11%)	11 163 (14%)	3319 (25%)	10 (9%)
unknown	5 (0%)	0 (0%)	0 (0%)	3 (0%)	2 (0%)	0 (0%)	0 (0%)
contact involves touch?							
no	117 614 (81%)	18 (12%)	7085 (47%)	28 729 (75%)	69 763 (88%)	11 929 (90%)	90 (78%)
yes	27 850 (19%)	133 (88%)	8065 (53%)	9343 (25%)	9056 (11%)	1228 (9%)	25 (22%)
unknown	196 (0%)	1 (1%)	35 (0%)	26 (0%)	92 (0%)	42 (0%)	0 (0%)
contact frequency							
<1 time per week	24 304 (17%)	2 (1%)	528 (3%)	6370 (17%)	15 977 (20%)	1425 (11%)	2 (2%)
1–3 times per week	15 370 (11%)	1 (1%)	533 (4%)	5675 (15%)	7972 (10%)	1182 (9%)	7 (6%)
4+ times per week	105 938 (73%)	149 (98%)	14 121 (93%)	26 040 (68%)	54 939 (70%)	10 583 (80%)	106 (92%)
unknown	48 (0%)	0 (0%)	3 (0%)	13 (0%)	23 (0%)	9 (0%)	0 (0%)
contact duration							
<10 minutes	50 158 (34%)	3 (2%)	1373 (9%)	12 742 (33%)	32 563 (41%)	3471 (26%)	6 (5%)
10–59 min	21 643 (15%)	13 (9%)	2137 (14%)	4981 (13%)	12 298 (16%)	2188 (17%)	26 (23%)
60+ min	73 830 (51%)	136 (89%)	11 672 (77%)	20 373 (53%)	34 028 (43%)	7538 (57%)	83 (72%)
unknown	29 (0%)	0 (0%)	3 (0%)	2 (0%)	22 (0%)	2 (0%)	0 (0%)

Total number of reported contacts stratified by group size, age of contact, setting in which the contact occurred, whether the contact involved touch, contact frequency and contact duration. Total contacts are further stratified by the age of the participant. Column percentages may not sum to exactly 100% due to rounding error.

**Figure 1.**
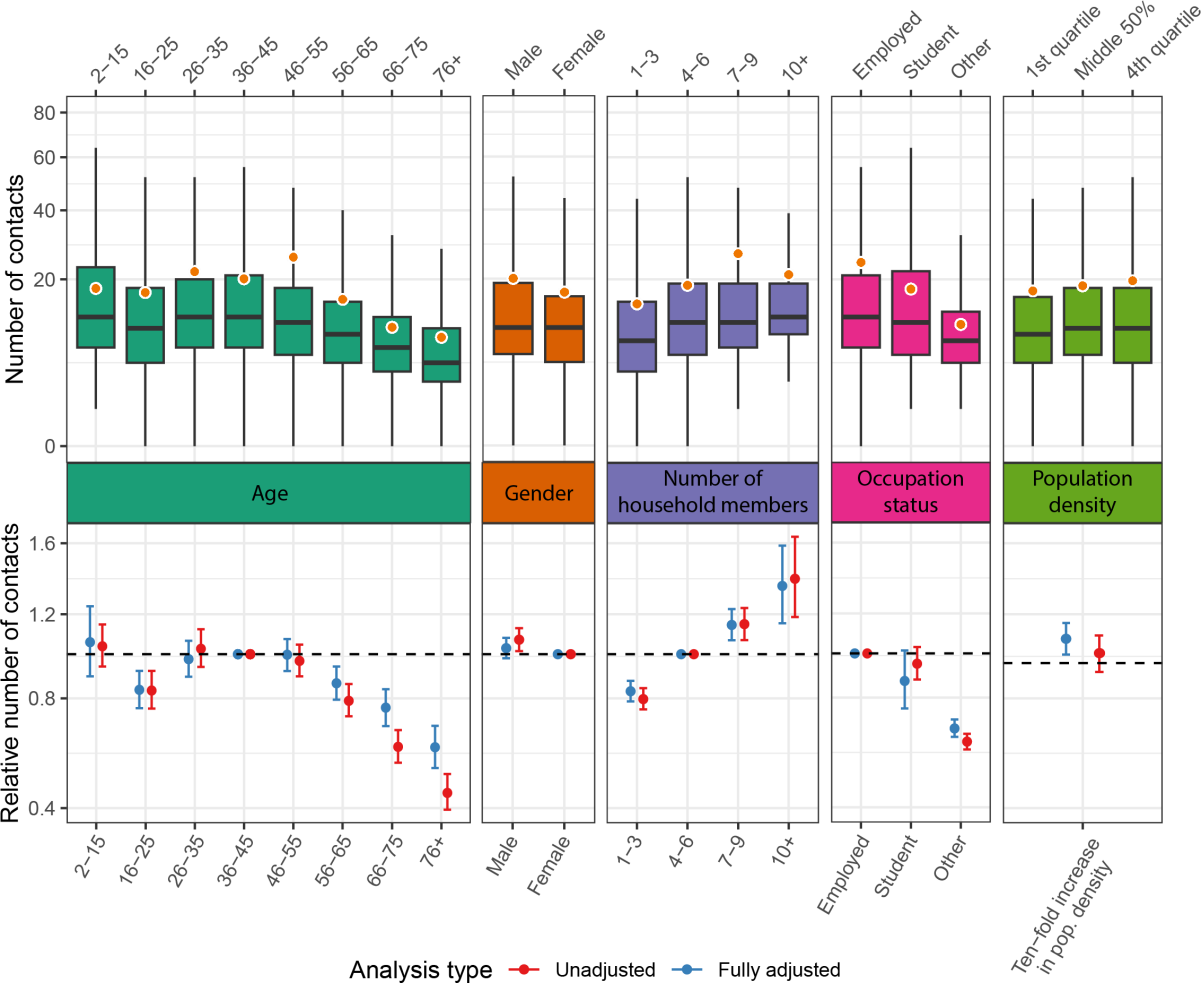
Variation in reported daily contacts by participant characteristics. Top panel: distribution of the number of all reported contacts stratified by age group, gender, household size, occupation status and population density. The box spans from the 25th to the 75th percentile, with the solid black line indicating the median. The whiskers extend to the largest and smallest values no more than 1.5 times the interquartile range from the hinge. The orange point shows the mean. Outliers are not shown. Bottom panel: the relative number of all reported contacts and 95% confidence intervals from unadjusted (red) and adjusted (blue) hierarchical quasi-Poisson regression. The fully adjusted model includes age, gender, household size, occupation status and log_10_ population density.

The total number of individual contacts remains relatively steady through childhood and into middle age and then appears to decline among older adults (figure 1). The median number of daily contacts ranged from 10 to 13 in age groups 55 and younger, compared with 6 in adults over age 65. These trends hold when restricting to contacts made outside the home (electronic supplementary material, figure S1).

After adjusting for other individual- and household-level characteristics, there remains a clear association between age and the total number of daily contacts (figure 1; electronic supplementary material, table S2). Compared with adults aged 36–45, children under 16 have a similar number of contacts (relative rate (RR): 1.06, 95% CI: 0.89–1.25), while those aged 16–25 have a lower number of contacts (RR: 0.84, 95% CI: 0.76–0.92). There are no significant differences from the reference group in daily contacts for adults aged 26–35 (RR: 0.98, 95% CI: 0.89–1.07; figure 1; electronic supplementary material, table S2). Rates of daily contact begin to decline after age 55, with those aged 56–65 having 14% fewer daily contacts (RR: 0.86, 95% CI: 0.79–0.94), those 66–75 having 24% fewer contacts (RR: 0.76, 95% CI: 0.69-0.84) and those aged 76+ having 40% fewer contacts (RR: 0.60, 95% CI: 0.53–0.69).

Many factors included in the model, such as employment status and household size, are strongly associated with age and probably mediate the relationship between age and the number of daily contacts; however, the age trend remains after adjustment (figure 1; electronic supplementary material, table S2). Employed individuals have the highest number of daily contacts, especially outside the home. Compared with those who are employed, students make 27% fewer extra-household contacts (RR: 0.73, 95% CI: 0.58–0.91), and unemployed or retired individuals make 51% fewer extra-household contacts (RR: 0.49, 95% CI: 0.45–0.53; figure 1; electronic supplementary material, table S2). Looking across all contacts, those in larger households have more contacts, and the number of daily contacts increases with population density (figure 1; electronic supplementary material, table S2). The impact of population density is more pronounced for extra-household contacts, where we see a significant increase in contacts occurring outside the home with increasing population density (RR: 1.20 per 10-fold increase in population density, 95% CI: 1.07–1.33; electronic supplementary material, figure S1 and table S2). The distribution of the number of daily contacts does not appear to differ significantly by gender.

Limiting to observations with complete covariate information and after trimming, there were 52 448 potential contact triangles across all four follow-up visits (2nd visit: 16 330; 3rd: 14 235; 4th: 12 018; 5th: 9865). Of these, 67% were connected (2nd visit: 66%; 3rd: 66%; 4th: 67%; 5th: 71%).

**Figure 2.**
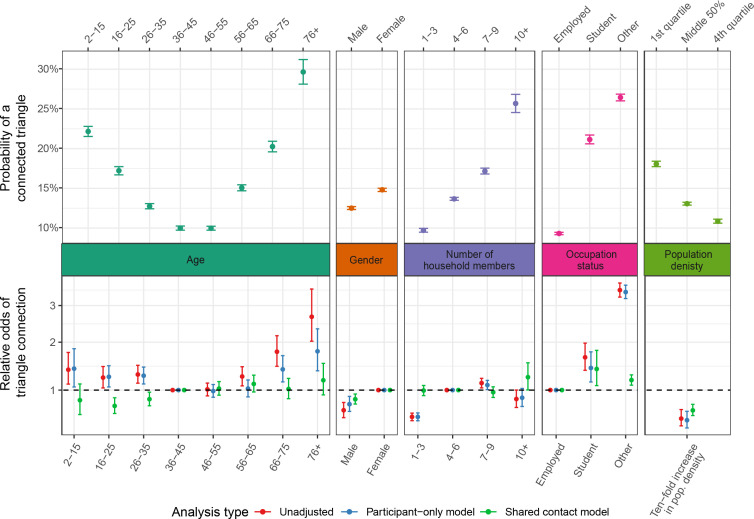
Variation in contact triangle connectedness by participant characteristics. Top panel: the expected probability of a contact triangle being connected stratified by participant characteristics for all contacts. Probabilities are obtained from unadjusted non-hierarchical logistic regression incorporating group size. Bottom panel: association between participant-level characteristics and odds of triangle connection for triangles involving all contacts. Estimates are from unadjusted models (red), models including only participant- and household-level characteristics (blue) and models including participant- and household-level characteristics, contact-level characteristics and indicators of shared characteristics between contacts (green).

The odds of potential triangles being connected follow a U-shaped curve by age, with the probability of triangles being connected ranging from 10 to 20% and hitting its lowest point among working adults aged 36–55 (figure 2). Those aged 2–15 have 1.38 (95% CI: 1.11–1.76) times the odds of being part of a connected triangle compared with those aged 36–45 (figure 2; electronic supplementary material, table S4), and those aged 76+ have 2.67 times the odds (95% CI: 2.03–3.51) on an unadjusted basis.

Our analysis suggests that the U-shaped pattern by age can be explained by the characteristics of the contacts themselves, such as the settings in which they occur. Adjusting for individual- and household-level characteristics has little impact on the relationship between key covariates, including age, and the odds of a triangle being connected (figure 2; electronic supplementary material, table S4). However, adjusting for contact characteristics (e.g. contact setting) alters the relationship between age and local clustering, eliminating the U-shaped pattern seen in univariate analyses. This suggests that age-specific contact characteristics, such as setting and intimacy, explain the majority of increased local clustering of contacts in younger individuals. Other patterns by gender, occupation and population density are mitigated but remain largely unchanged (figure 2; electronic supplementary material, table S4).

Being female, living in a large household, and being a student or unemployed/retired were also associated with increased local clustering (i.e. a higher probability of potential triangles being connected). Local clustering was also higher in more rural communities, with a 0.57-fold (95% CI: 0.48–0.69) decrease in the odds of a triangle being connected for each 10-fold increase in population density (figure 2; electronic supplementary material, table S4).

**Figure 3.**
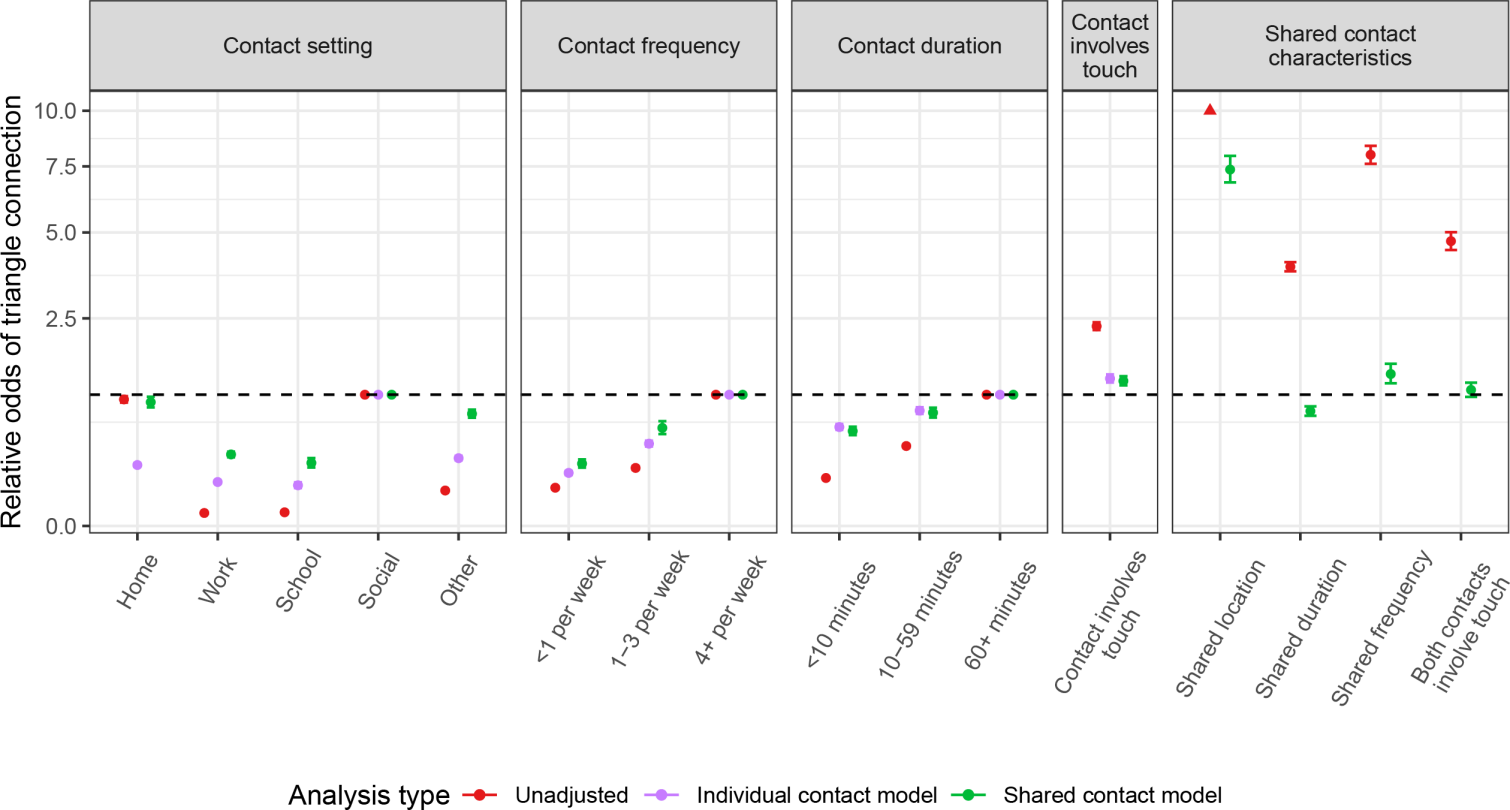
Association between contact-level characteristics and odds of triangle connection for triangles involving all contacts. Estimates from unadjusted models (red), models including only participant-, household-level and contact-level characteristics (purple) and models including participant- and household-level characteristics, contact-level characteristics and indicators of shared characteristics between contacts (green) are shown. The triangle at the upper plot limit indicates an estimate that is greater than 10.0.

**Figure 4.**
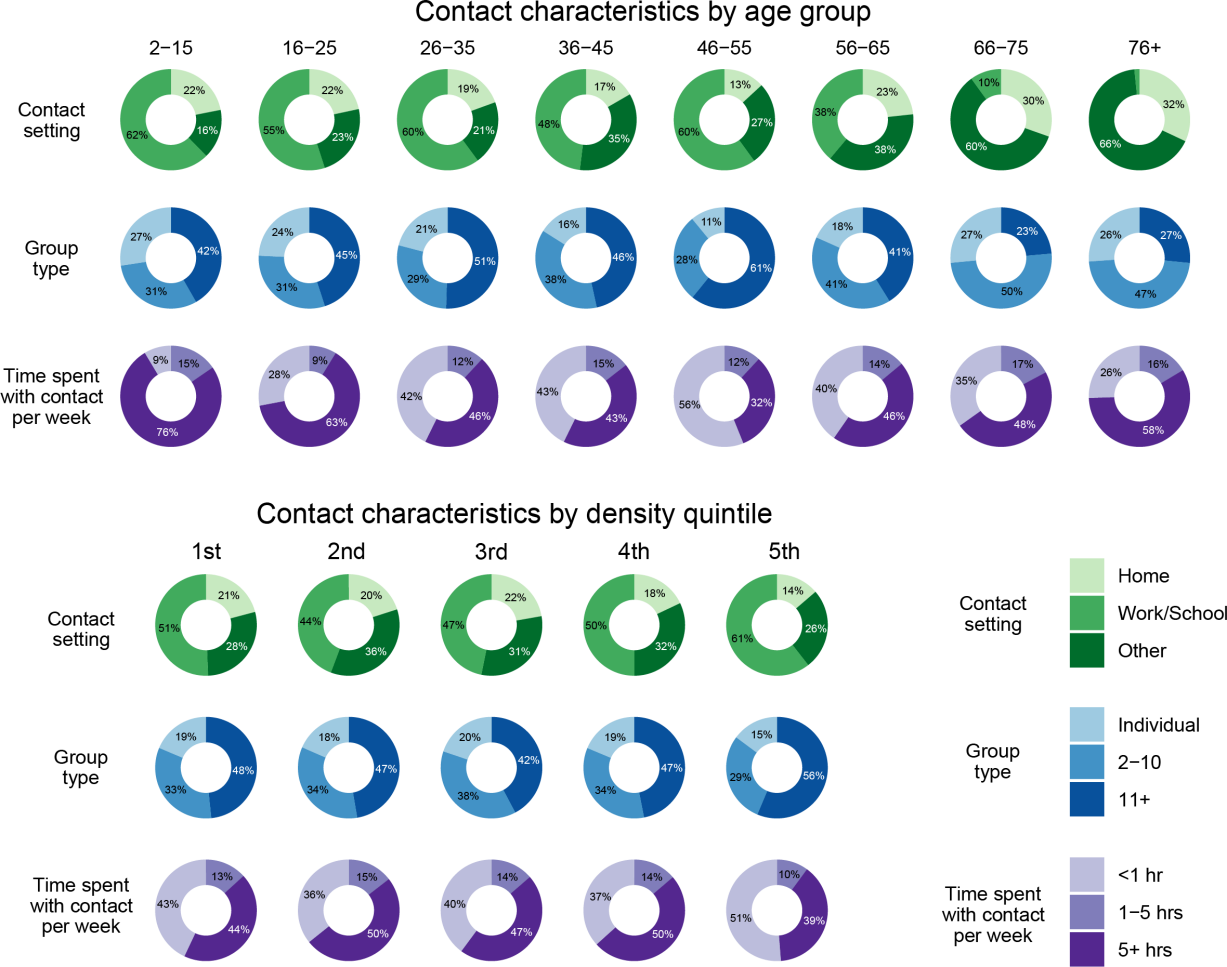
Decomposition of the total number of contacts by contact setting, group size and weekly time spent with contact by age group and population density quintile. Percentages may not sum to exactly 100% due to rounding error.

When we examine the nature of the contacts themselves, more intimate contacts—those that are higher duration, occur more frequently, or involve touch—are more likely to be part of a connected triangle (figure 3; electronic supplementary material, table S4). Such high-intimacy contacts make up a larger proportion of contacts in the youngest and oldest age groups as compared with working-age adults (figure 4; electronic supplementary material, table S5). In the shared contact model, which includes all participant-, household- and contact-level characteristics, as well as indicators for shared characteristics between the contacts, potential triangles involving contacts that take place in a social setting are most likely to be connected, while those involving school or work contacts are least likely to be connected (odds ratio (OR): 0.23, 95% CI: 0.20–0.27 for school versus social and OR: 0.30, 95% CI: 0.27–0.32 for work versus social; figure 3; electronic supplementary material, table S4). This contributes to, but does not fully explain, the increased levels of clustering observed in older adults, for whom most contacts occur in social or other settings (figure 4). If the two contacts in the potential triangle occur in the same setting (e.g. both at work or both in a social setting), they have 7.37 times (95% CI: 6.85–7.95) the odds of forming a connected triangle (electronic supplementary material, table S4).

Sensitivity analyses show that these associations are robust to differing certainty cut-offs for defining a triangle as connected. In adjusted analyses, the elimination of the U-shaped pattern with age after adjustment for contact characteristics, as well as the significant associations of sex, population density, occupation status, contact setting, duration and intimacy, all remained under varying levels of this assumption. Overall, a model that accounts for participant-level as well as individual- and shared contact-level characteristics most parsimoniously explains the data, as measured by WAIC (electronic supplementary material, table S6).

Accounting for the underlying triangles implied by groups expands the total number of potential triangles to 409 397. Contacts reported as groups are less likely to be part of a connected triangle. In the shared contact model, the presence of a group contact in a potential triangle, regardless of group size, reduces the odds of that triangle being connected by 74% (OR: 0.26, 95% CI: 0.24–0.28) and each additional person in a group results in a further 63% reduction (OR: 0.37, 95% CI: 0.36–0.38) in the odds of triangle connectedness (electronic supplementary material, table S4). The differential effect of group contacts is probably driven by fundamental differences in the types of individuals who make up a group contact and how such contacts are reported. Group contacts tend to be of shorter duration and lower frequency than individual contacts and are more likely to occur in the workplace, indicating such contacts are less intimate. The presence of large groups is a driver of much of the low levels of clustering observed in the contacts of middle-aged participants (figure 4; electronic supplementary material, figure S4).

## 4. Discussion

We found distinct patterns in the number and clustering of contacts by age, population density and contact type in a detailed analysis of contact networks in southern China. Among both older individuals and those in less dense areas, the number of contacts is reduced, and the chance that those contacts themselves know each other is increased (i.e. there is higher local clustering). More intimate contacts are also more likely to know other contacts.

**Figure 5.**
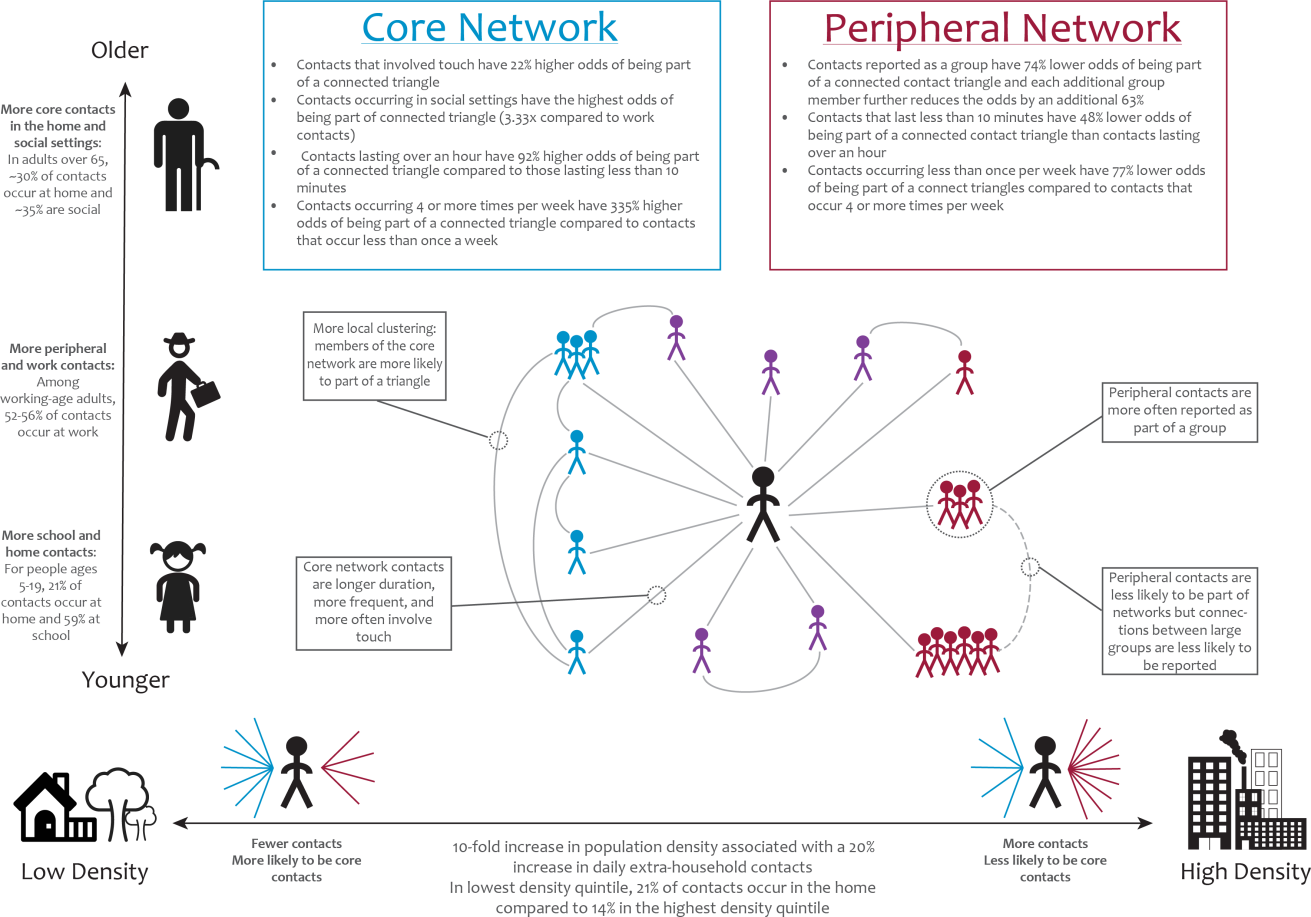
Framework for the decomposition of contact network into core and peripheral contacts. Core contacts tend to occur more frequently, be longer in duration and be more locally clustered, whereas peripheral contacts are less clustered and more likely to be reported as part of large groups. Heterogeneity in network structure across age groups and between urban and rural settings may be explained by differences in the percentage of individuals’ networks composed of core versus peripheral contacts.

Based on these results, we propose a framework for thinking about the structure of contact networks wherein an individual’s contact network can be decomposed into ‘core’ and ‘peripheral’ sub-networks (figure 5). Core contacts are more intimate, tending to be longer duration, more frequent and more often involving touch, whereas peripheral contacts are less intimate and more likely to be reported as part of a group. Compared with peripheral contacts, core contacts are more locally clustered. Within this framework, differences in contact behaviour between individuals and communities can be explained in part by variation in the number of core and peripheral contacts made. While we believe the existence of these categories is a more parsimonious explanation of our results, further exploration of this hypothesis, including the establishment of strict definitions that allow for the categorization of contacts as core versus peripheral, is an area for future work.

The combined effect of local clustering and network degree on disease transmission will vary within communities. Prior work has shown that increased contact duration is associated with increased infection risk for pathogens such as SARS-CoV-2 [29] and influenza [30]. This indicates that core contacts pose a higher per contact risk of onward transmission compared with peripheral contacts, which are more likely to be workplace contacts. Older adults and those living in rural areas tend to have fewer contacts but higher levels of local clustering, indicating that core contacts make up a greater percentage of these individuals’ daily contacts. This implies that a pathogen may be less likely to be introduced, but more likely to spread quickly within the local network of individuals dwelling in rural areas once introduction does take place. In contrast, working-age adults have more lower intensity peripheral contacts and, therefore, a higher number of daily contacts but lower levels of clustering among those contacts. For these individuals, exposure to pathogens circulating outside one’s core network may be more frequent, but transmission may not be as intense within one’s local network.

Fully understanding the impact of network structure on pathogen transmission dynamics would require full enumeration of the underlying contact network, which has largely been the gold standard approach for measuring social contact behaviour. However, this is generally not possible at scale for the large variety of contacts relevant to respiratory pathogen transmission. Prior contact surveys aiming to capture such contacts have emphasized measuring first-order contact structure [9–12]. These results have informed assumptions of how transmission may occur throughout these networks; however, this approach neglects to account for higher order structures that may significantly alter patterns of pathogen spread. Our work represents an intermediate approach wherein relative metrics of a second-order network structure can be estimated in the absence of a fully enumerated network. Such an approach can be used in contact survey efforts globally to improve the understanding of how contact network structure, and therefore transmission dynamics on these networks, vary within and between regions.

Prior work investigating heterogeneity in contact behaviour has emphasized the variations in the number and characteristics of first-order contacts across age groups. Studies have shown that the number of contacts is highest in school-age children and lowest in older adults, and that contacts tend to be assortative by age [9,11,12,23]. Given the association between age and other characteristics relevant to contact behaviour, such as occupational status and household structure, it is possible that much of the observed age-related variation is mediated by these more proximal factors. Our analysis demonstrates that even after adjustment for these mediating factors, a strong association remains between age and the number of contacts as well as age and local clustering; however, the shape of these relationships does change. Furthermore, the adjusted association between age and the number of contacts observed in this study is consistent with studies in other Asian settings [23,30,31] but deviates from that observed in many prior surveys of European populations [11], with school-age children having similar numbers of contacts to working-age adults.

Further, previous studies often suffer from truncation issues, wherein individual contacts are too numerous to report individually [11]. This is especially important for working-age adults who may encounter many people in the workplace whom they do not know personally. The use of group contacts helps to mitigate this issue. However, this approach is also subject to several limitations. Group contacts may be fundamentally different from contacts reported individually, with such contacts tending to last for shorter amounts of time and reoccur less frequently. As less information is generally available about the individuals composing a group contact, the inferences that can be made about groups are more limited. We are unable to analyse the association between network properties and contacts’ age when including groups, as a single group may contain individuals of many ages.

When assessing local clustering, the definition of contact used for individual and group contacts differs. Participants are asked whether at least one member of the group knows another reported contact. When groups are reported as being in a connected triangle, we can only know that at least one or more of the many potential triangles involving each individual in the group is connected. Therefore, for groups in a connected triangle, as group size increases, the information contribution of these observations decreases. Participants’ uncertainty about relationships between contacts also increases as group size increases. We therefore find that the observed connectivity of triangles involving groups is less than what would be expected based on the size of the group(s) involved. Given that large group contacts are more likely to occur in certain settings, such as the workplace, it is not possible to fully disentangle the impact of group size and setting.

Contacts are self-reported and therefore subject to recall bias. This bias may be differential across participants, especially with regard to age, as a parent must report contacts on their child’s behalf. This study uses a structured interview process when administering the contact survey, which has been shown to improve recall [32]. The data in this study were collected over four follow-up visits spanning seven years. The turnover in study staff and other temporal trends may therefore create systematic variation between visits. We address this concern in part by including visit-level fixed effects in our models.

The FluScape study is a large, representative sample of a highly populous area in southern China and utilizes a definition of contact applicable to a broad variety of respiratory pathogens. While the generalizability of these results to locations with very different population structures may be limited, much of the world’s population lives in this region, and the population structure of southern China is reflective of many areas throughout the region. The applicability of these results to pathogens transmitted via other modes (for example, sexual contact) may also be limited. Additionally, although children under 5 may play a key role in transmission dynamics [22] and are under-represented in this study, the transition to school is an important driver of the differential contact behaviour of children compared with adults. We expect that contact behaviour driven by school attendance is still well captured in this study as primary school entry generally occurs after age 5.

This study characterizes variation in first- and second-order contact network structures among individuals and communities in southern China and identifies key correlates of this variation, in particular age and population density. Quantification of both the number of contacts individuals make and the local clustering among those contacts is required to more fully assess the risk of pathogen introduction and onward spread within community sub-groups. Results from contact surveys as well as facility-based measurements using sensors have been used to parametrize contact networks for numerous large simulation models [33,34]. However, many routinely used models assume all contacts are equal and do not incorporate higher order network structure. Prior work has shown that many aspects of network structure can be captured by modelling the number of triangles within the network [2,35,36], one of the outcomes directly measured in this study. An approach that incorporates the distinction between core and peripheral contacts may also allow for more effective targeting of measures such as social distancing (for example, the least disruptive social distancing measures probably target peripheral contacts). Future work is needed to develop methods to best use this information in mechanistic models of infectious disease transmission, with the goal of yielding models that will better inform prevention and control efforts.

## Data Availability

The data used in the analysis and analytic code are available at [37]. Supplementary material is available online [38].

## References

[1] Eames K, Bansal S, Frost S, Riley S. 2015 Six challenges in measuring contact networks for use in modelling. Epidemics **10**, 72–77. (10.1016/j.epidem.2014.08.006) 25843388

[2] Pellis L, Ball F, Bansal S, Eames K, House T, Isham V, Trapman P. 2015 Eight challenges for network epidemic models. Epidemics **10**, 58–62. (10.1016/j.epidem.2014.07.003) 25843385

[3] Keeling MJ, Eames KTD. 2005 Networks and epidemic models. J. R. Soc. Interface **2**, 295–307. (10.1098/rsif.2005.0051) 16849187 PMC1578276

[4] Leung NHL. 2021 Transmissibility and transmission of respiratory viruses. Nat. Rev. Microbiol. **19**, 528–545. (10.1038/s41579-021-00535-6) 33753932 PMC7982882

[5] Fournet J, Barrat A. 2014 Contact patterns among high school students. PLoS One **9**, e107878. (10.1371/journal.pone.0107878) 25226026 PMC4167238

[6] Vanhems P, Barrat A, Cattuto C, Pinton JF, Khanafer N, Régis C, Kim B-a, Comte B, Voirin N. 2013 Estimating potential infection transmission routes in hospital wards using wearable proximity sensors. PLoS One **8**, e73970. (10.1371/journal.pone.0073970) 24040129 PMC3770639

[7] Helleringer S, Kohler HP. 2007 Sexual network structure and the spread of HIV in Africa: evidence from Likoma Island, Malawi. AIDS **21**, 2323–2332. (10.1097/qad.0b013e328285df98) 18090281

[8] Kutter JS, Spronken MI, Fraaij PL, Fouchier RA, Herfst S. 2018 Transmission routes of respiratory viruses among humans. Curr. Opin. Virol. **28**, 142–151. (10.1016/j.coviro.2018.01.001) 29452994 PMC7102683

[9] Mousa A *et al*. 2021 Social contact patterns and implications for infectious disease transmission – a systematic review and meta-analysis of contact surveys. eLife **10**, 70294. (10.7554/elife.70294) PMC8765757 34821551

[10] Hoang T, Coletti P, Melegaro A, Wallinga J, Grijalva CG, Edmunds JW, Beutels P, Hens N. 2019 A systematic review of social contact surveys to inform transmission models of close-contact infections. Epidemiology **30**, 723–736. (10.1097/ede.0000000000001047) 31274572 PMC6684224

[11] Mossong J *et al*. 2008 Social contacts and mixing patterns relevant to the spread of infectious diseases. PLoS Med. 0381–0391. **5**, e74. (10.1371/journal.pmed.0050074) PMC2270306 18366252

[12] Béraud G *et al*. 2015 The French connection: the first large population-based contact survey in France relevant for the spread of infectious diseases. PLoS One 1–22. **10**, e0133203. (10.1371/journal.pone.0133203) PMC4503306 26176549

[13] Eames KTD, Keeling MJ. 2002 Modeling dynamic and network heterogeneities in the spread of sexually transmitted diseases. Proc. Natl Acad. Sci. USA **99**, 13330–13335. (10.1073/pnas.202244299) 12271127 PMC130633

[14] Volz EM, Miller JC, Galvani A, Ancel Meyers L. 2011 Effects of heterogeneous and clustered contact patterns on infectious disease dynamics. PLoS Comput. Biol. **7**, e1002042. (10.1371/journal.pcbi.1002042) 21673864 PMC3107246

[15] Badham J, Stocker R. 2010 The impact of network clustering and assortativity on epidemic behaviour. Theor. Popul. Biol. **77**, 71–75. (10.1016/j.tpb.2009.11.003) 19948179

[16] Haw DJ, Pung R, Read JM, Riley S. 2020 Strong spatial embedding of social networks generates nonstandard epidemic dynamics independent of degree distribution and clustering. Proc. Natl Acad. Sci. USA **117**, 23636–23642. (10.1073/pnas.1910181117) 32900923 PMC7519285

[17] Britton T, Deijfen M, Lagerås AN, Lindholm M. 2008 Epidemics on random graphs with tunable clustering. J. Appl. Probab. **45**, 743–756. (10.1239/jap/1222441827)

[18] Bansal S, Grenfell BT, Meyers LA. 2007 When individual behaviour matters: homogeneous and network models in epidemiology. J. R. Soc. Interface **4**, 879–891. (10.1098/rsif.2007.1100) 17640863 PMC2394553

[19] Eames KTD, Keeling MJ. 2003 Contact tracing and disease control. Proc. R. Soc. Lond. B **270**, 2565–2571. (10.1098/rspb.2003.2554) PMC1691540 14728778

[20] Read JM, Keeling MJ. 2003 Disease evolution on networks: the role of contact structure. Proc. R. Soc. Lond. B **270**, 699–708. (10.1098/rspb.2002.2305) PMC1691304 12713743

[21] Read JM, Lessler J, Riley S, Wang S, Tan LJ, Kwok KO, Guan Y, Jiang CQ, Cummings DAT. 2014 Social mixing patterns in rural and urban areas of southern China. Proc. R. Soc. B **281**, 20140268. (10.1098/rspb.2014.0268) PMC4024290 24789897

[22] Jackson C, Vynnycky E, Hawker J, Olowokure B, Mangtani P. 2013 School closures and influenza: systematic review of epidemiological studies. BMJ Open **3**, e002149. (10.1136/bmjopen-2012-002149) PMC3586057 23447463

[23] Kwok KO, Cowling B, Wei V, Riley S, Read JM. 2018 Temporal variation of human encounters and the number of locations in which they occur: a longitudinal study of Hong Kong residents. J. R. Soc. Interface **15**, 20170838. (10.1098/rsif.2017.0838) 29367241 PMC5805989

[24] Jiang CQ *et al*. 2017 Cohort profile: a study of influenza immunity in the urban and rural Guangzhou region of China: the Fluscape study. Int. J. Epidemiol. **46**, dyv353. (10.1093/ije/dyv353) PMC6251537 26875566

[25] Lebakula V *et al*. 2022 LandScan global 30 arcsecond annual global gridded population datasets from 2000 to 2022. Sci. Data **12**, 495. (10.1038/s41597-025-04817-z) PMC11933676 40128284

[26] Wedderburn RWM. 1974 Quasi-likelihood functions, generalized linear models, and the Gauss—Newton method. Biometrika **61**, 439–447. (10.1093/biomet/61.3.439)

[27] Stan Development Team. 2019 Stan users guide and reference manual, version 2.21. See https://mc-stan.org.

[28] Gelman A, Rubin DB. 1992 Inference from iterative simulation using multiple sequences. Stat. Sci. **7**, 457–511. (10.1214/ss/1177011136)

[29] Ferretti L *et al*. 2024 Digital measurement of SARS-CoV-2 transmission risk from 7 million contacts. Nature **626**, 145–150. (10.1038/s41586-023-06952-2) 38122820 PMC10830410

[30] Kwok KO, Cowling BJ, Wei VWI, Wu KM, Read JM, Lessler J, Cummings DA, Peiris JSM, Riley S. 2014 Social contacts and the locations in which they occur as risk factors for influenza infection. Proc. R. Soc. B **281**, 20140709. (10.1098/rspb.2014.0709) PMC4100506 25009062

[31] Leung K, Jit M, Lau EHY, Wu JT. 2017 Social contact patterns relevant to the spread of respiratory infectious diseases in Hong Kong. Sci. Rep. **7**, 7974. (10.1038/s41598-017-08241-1) 28801623 PMC5554254

[32] Mosser AE, Evans JR. 2019 Increasing the number of contacts generated during contact tracing interviews. Memory **27**, 495–506. (10.1080/09658211.2018.1529247) 30295153

[33] Machens A, Gesualdo F, Rizzo C, Tozzi AE, Barrat A, Cattuto C. 2013 An infectious disease model on empirical networks of human contact: bridging the gap between dynamic network data and contact matrices. BMC Infect. Dis. **13**. (10.1186/1471-2334-13-185) PMC3640968 23618005

[34] Potter GE, Handcock MS, Longini IM, Halloran ME. 2012 Estimating within-school contact networks to understand influenza transmission. Ann. Appl. Stat. **6**, 1–26. (10.1214/11-AOAS505) 22639701 PMC3359895

[35] Pellis L, House T, Keeling MJ. 2015 Exact and approximate moment closures for non-Markovian network epidemics. J. Theor. Biol. **382**, 160–177. (10.1016/j.jtbi.2015.04.039) 25975999

[36] House T, Keeling MJ. 2011 Insights from unifying modern approximations to infections on networks. J. R. Soc. Interface **8**, 67–73. (10.1098/rsif.2010.0179) 20538755 PMC3024819

[37] Smith CP. 2025 UNCIDD/fluscape-contacts: version 1.0 (v1.0.0). Zenodo (10.5281/zenodo.17195348)

[38] Smith CP, Read JM, Riley S, Cummings D, Kwok KO, Jiang CQ *et al*. 2025 Supplementary material from: First and Second-Order Social Contact Network Structure in Southern China. Figshare. (10.6084/m9.figshare.c.8096965)41290138

